# High Pressure Compression-Molding of α-Cellulose and Effects of Operating Conditions

**DOI:** 10.3390/ma6062240

**Published:** 2013-05-30

**Authors:** Thibaud Pintiaux, David Viet, Virginie Vandenbossche, Luc Rigal, Antoine Rouilly

**Affiliations:** 1 Université de Toulouse, INP-ENSIACET, LCA (Laboratoire de Chimie Agro industrielle), Toulouse F 31030, France; E-Mails: virginie.vandenbossche@ensiacet.fr (V.V.); luc.rigal@ensiacet.fr (L.R.); antoine.rouilly@ensiacet.fr (A.R.); 2 INRA, UMR 1010 CAI, Toulouse F 31030, France; 3 The Green Factory, 27 rue Chanez, Paris 75016, France; E-Mail: david.viet@ensiacet.fr

**Keywords:** α-cellulose, compression-molding, agromaterials, biomaterials, mechanical properties

## Abstract

Commercial α-cellulose was compression-molded to produce 1A dog-bone specimens under various operating conditions without any additive. The resulting agromaterials exhibited a smooth, plastic-like surface, and constituted a suitable target as replacement for plastic materials. Tensile and three-points bending tests were conducted according to ISO standards related to the evaluation of plastic materials. The specimens had strengths comparable to classical petroleum-based thermoplastics. They also exhibited high moduli, which is characteristic of brittle materials. A higher temperature and higher pressure rate produced specimens with higher mechanical properties while low moisture content produced weaker specimens. Generally, the strong specimen had higher specific gravity and lower moisture content. However, some parameters did not follow the general trend e.g., thinner specimen showed much higher Young’s Modulus, although their specific gravity and moisture content remained similar to control, revealing a marked skin-effect which was confirmed by SEM observations.

## 1. Introduction

The agromaterials, greatly reviewed by Rouilly and Rigal in 2002 are a very promising field, effective in reducing green-house effect (few or no petroleum use) and producing easily biodegradable products [1]. Using only agricultural wastes instead of dedicated crops is tomorrow's challenge for producing such materials because of the land-usage competition between food in one hand, and industry in the other hand (fuel, plastic-like materials, solvents, *etc.*). Under this consideration, researchers have been working on the molding of natural fiber materials without addition of any glue, binder, or polymeric resin.

The major part of these reports is about binderless particleboards (historically Mobarak *et al*. [2] 1982, Suchsland *et al*. [3] 1987, Suzuki *et al*. [4] 1998, Anglès *et al*. [5] 1999, Okuda and Sato [6] 2004, Van Dam *et al*. [7] 2004, Widyorini *et al*. [8] 2005 followed by many others) which use low pressure molding (typically around 10 MPa) and thermo-triggered self-binding ability of natural fibers, with help of a steam treatment, or not.

High pressure molding (generally over 100 MPa) in two or three dimensions and extrusion have been more recently studied on pure non modified natural fibers (e.g., Miki *et al*. [9] 2003, Imanishi *et al*. [10] 2005, Yamashita *et al*. [11] 2007, Rouilly *et al*. [12] 2012).

These papers explored the conditions of molding and their effect on the mechanical properties of the resulting materials, but in these cases, since the starting material contains cellulose, hemicelluloses, lignin and also proteins or short polysaccharides, the contribution of each component to the bonding remains unclear. It is widely admitted and reported that the self-binding ability of natural fibers is mostly based on melting/glass transition and degradation products of lignin and hemicelluloses. In addition, for materials that contain protein and sugars, their presence can have a major role. The main component of every plant material is cellulose, it is therefore of major interest to study the self binding ability of pure cellulose to produce agromaterials.

Cellulose can be processed into very high performance films and sheets according to the concepts of “all cellulose composite” and “nanofibers paper”. The first concept uses a sequential exchange of solvent from water to DMAc through ethanol and acetone and the final addition of LiCl to solubilize cellulose. Then removing the solvent by rinsing and casting produces very high performance sheets: e.g., Nishino *et al*. [13] processed a composite made from pre-oriented ramie fibers and Kraft pulp solubilized cellulose matrix which obtained a dynamic storage modulus of 45 GPa and an amazing tensile strength of 480 MPa. Gindl and Keckes [14] processed micro-crystalline cellulose the same way and obtained an anisotropic all cellulose film which had 13 GPa of Young’s modulus and 243 MPa of tensile strength.

These amazing performances seemed to be possible only with the dissolution of cellulose using hazardous chemicals but Yano *et al*. [15] reported the production of high strength material without solubilization of fibers: working with Kraft pulp, they used repeated high pressure homogenizer treatments to reduce the size of the cellulose microfibrils to a nanometer scale, which was called nanofibers. The suspension was concentrated with cold pressing, followed by an air drying step and a final hot pressing step. They obtained 16 GPa of bending modulus and a high bending strength of 250 MPa. Iwamoto *et al*. [16] reported 8 GPa of Young’s modulus and 90 MPa of tensile strength using a special grinding treatment of cellulose water suspension and a similar process of filtering and drying. Finally, Nogi *et al*. [17] presented an optically transparent material based on the findings of Yano *et al*. and the grinding treatment of Abe *et al*. [18] which had a Young’s modulus of 13 GPa and a tensile strength of 223 MPa.

Compression-molding as an alternative method to time consuming air drying process was also studied. Nilsson *et al*. [19] reported the production of compression molded material from wet cellulose disintegrated pulps using a two-stage (Cold, 7 min, 0.6 MPa/Hot, 20 min,−45 MPa) pressing step, their material reached 11 GPa of Young’s modulus and 76 MPa of tensile strength. Also, Rampinelli *et al*. [20] studied the mechanical properties of compression molded highly refined micro-fibrillated cellulose. They produced a material with 180 MPa of tensile strength, 165 MPa of bending strength and a bending modulus of 9.4 GPa with an optimized process using 120 MPa of pressure, for 6 min at 160 °C. Finally, Zhang *et al*. [21] produced ball-milled cellulose material from non treated cotton linters micro-crystalline cellulose using a high shear device usually dedicated to the sintering of metals and reported a bending modulus of 1.84 GPa using DMA measurements.

The pharmaceutical tablet production uses cellulose as excipient, but it uses room temperature to prevent degradation of the active substances and a lot of additives can be used [22].

This paper examines the possibility of molding purified cellulose under its most current and simple form, α-cellulose, non refined, untreated, unmodified and without the use of a water suspension step. The goal of this project is to produce 3D objects with a fast and eco-friendly yet economically viable process of compression molding of raw lignocellulosic materials. Molding parameters were deeply explored on α-cellulose as the major polymer in lignocellulosic materials. The effects of the parameters on the mechanical properties of the molded samples were investigated. The three-points bending and tensile strengths and moduli, specific density and moisture content of the compression-molded specimen were measured and results were discussed.

## 2. Results

### 2.1. Macroscopic Observations of the Compressed Specimens

**Figure 1:**
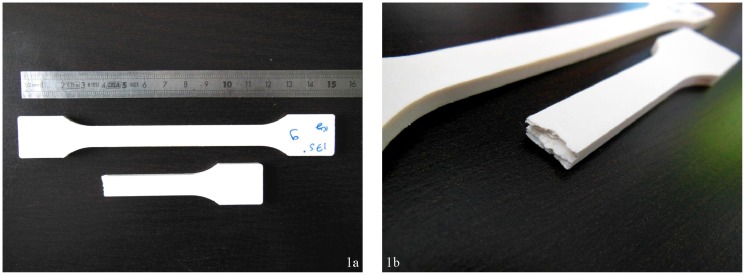
**(a)** α-cellulose compression-molded specimen; **(b)** Bending fracture where the laminated structure can be observed.

The surface of the compressed material exhibited a smooth, plastic-like surface (Figure 1a). The majority of the specimens had a slightly yellow-grey color, compared to the starting material. The 200 °C specimens were looking more yellow and the 0% moisture content looked whiter, similar to the starting material. 

Breaking a specimen revealed a laminated inner structure (Figure 1b). The material consisted in thin layers, perpendicular to the compression. On the 200 °C of pressing temperature condition, about one third of the specimens exhibited cracks between one or several layers, leading on extreme cases to the opening of the whole piece in two, often in the middle of the material. This phenomenon called delamination was previously observed in fibers molding when attempting to mold at high temperature [8,10] and also in the pharmaceutical tablets industry [22] although the molding of tablets is made at room temperature.

### 2.2. Effect of Mass Introduced in the Mold

5, 7.5, 10 (control) and 12.5 grams of α-cellulose were charged in the mold, and control press cycle (Figure 13, experimental section) was conducted to form the dog-bone specimen. This produced specimens of an average thickness of 1.92 ± 0.10, 2.79 ± 0.10, 3.61 ± 0.13 and 4.48 ± 0.14 mm for respectively 5, 7.5, 10 and 12.5 grams. Homogeneity was hard to obtain on thinnest specimens. Additional repetitions were consequently needed to obtain seven correct required specimens.

Control conditions produced specimen with very smooth surface, with a warm and plastic-like touch. Globally, the compression molded specimens had high mechanical properties, high moduli, very little elasticity and no plastic deformation (glassy material) compared to classical thermoplastic polymers. Mechanical properties of the control specimens were bending strength of 40.2 ± 2.3 MPa, bending modulus of 6.18 ± 0.21 GPa, tensile strength of 21.2 ± 1.1 MPa, and Young's modulus of 1.60 ± 0.03 GPa (Table 1).

**Figure 2:**
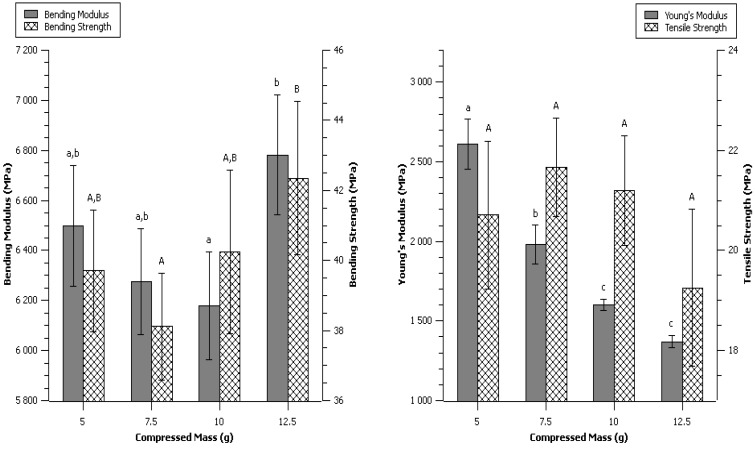
Effect of initial Compressed Mass. Data are given as mean ± confident interval (95%). The letters on top of the column express significant difference of means according to Scheffé test with a 95% confident level. Small letters for moduli and capital letters for strengths.

Lower or higher mass charged in the mold did not change significantly either the maximum strength at break in tensile or bending (Figure 2). Neither was the bending modulus altered. However, the Young’s modulus appeared drastically increased while mass was reduced. In fact, dividing the initial mass by two (from 10 to 5 g) led to a 63% increase of the Young’s Modulus.

**Table 1:** Summary of operating conditions and measurements made on α-cellulose compressed specimens. Values are represented with their means ± confident interval (95%). Seven to 15 replicates for the mechanical properties, 6 for determination of specific gravity and moisture content at 60% relative humidity and 25 °C.

Operating conditions/starting material						Compression-molded specimens																	
Pressure (MPa)	Temperature (°C)	Time (s)	Initial Mass (g)	Pressure rate (bar/s)	Relative Humidity of equilibrium	Bending Strength at break (MPa)			Bending Modulus (GPa)			Tensile Strength at break (Mpa)			Young's Modulus (Gpa)			Specific Gravity (g/mL)			Moisture Content at 60% RH 25 °C (%)		
**265**	**150**	**120**	**10**	**10**	**60%**	**40.2**	**±**	**2.3**	**6.18**	**±**	**0.21**	**21.2**	**±**	**1.1**	**1.60**	**±**	**0.03**	**1.503**	**±**	**0.006**	**7.02**	**±**	**0.05**
133	150	120	**5**	10	60%	39.7	±	1.7	6.50	±	0.24	20.7	±	1.5	2.61	±	0.16	1.502	±	0.006	7.02	±	0.08
			**7.5**			38.1	±	1.5	6.28	±	0.21	21.7	±	1.0	1.98	±	0.12	1.503	±	0.006	6.99	±	0.07
			**12.5**			42.4	±	2.2	6.78	±	0.24	19.3	±	1.6	1.37	±	0.04	1.503	±	0.006	7.11	±	0.05
**133**			10			37.8	±	2.1	5.78	±	0.22	18.6	±	1.1	1.67	±	0.03	1.503	±	0.003	7.01	±	0.09
	**100**					29.8	±	2.4	4.80	±	0.35	16.6	±	0.8	1.52	±	0.05	1.486	±	0.006	7.47	±	0.05
265	**100**					34.7	±	2.5	5.39	±	0.19	20.1	±	1.3	1.55	±	0.04	1.490	±	0.006	7.56	±	0.07
	**125**					37.3	±	2.0	5.62	±	0.17	20.0	±	0.9	1.55	±	0.06	1.505	±	0.003	7.22	±	0.02
	**175**					39.9	±	1.8	6.19	±	0.13	21.1	±	0.8	1.59	±	0.04	1.510	±	0.002	6.87	±	0.02
	**200**					43.2	±	3.0	7.17	±	0.31	22.4	±	1.2	1.73	±	0.09	1.513	±	0.006	6.86	±	0.18
	150	**3**				35.5	±	1.1	6.38	±	0.10	18.4	±	0.6	1.60	±	0.00	1.494	±	0.004	7.51	±	0.05
		**30**				39.8	±	1.2	5.80	±	0.19	20.0	±	1.0	1.65	±	0.06	1.506	±	0.003	7.02	±	0.03
		**300**				36.4	±	2.6	5.97	±	0.26	20.7	±	1.1	1.59	±	0.07	1.508	±	0.004	6.9	±	0.02
		120		**1**		31.5	±	2.6	4.93	±	0.53	18.0	±	1.5	1.56	±	0.05	1.507	±	0.004	6.93	±	0.05
				**2**		35.0	±	1.2	5.56	±	0.23	19.5	±	0.9	1.60	±	0.05	1.506	±	0.002	6.95	±	0.01
				**50**		41.3	±	2.0	6.03	±	0.37	21.9	±	0.8	1.61	±	0.06	1.499	±	0.007	7.08	±	0.05
				10	**0%**	15.7	±	1.5	4.02	±	0.63	8.8	±	0.4	1.30	±	0.04	1.489	±	0.003	7.62	±	0.02
					**45%**	36.8	±	2.7	5.61	±	0.41	20.0	±	1.0	1.60	±	0.05	1.497	±	0.004	7.17	±	0.08
					**75%**	32.4	±	0.9	5.30	±	0.18	17.0	±	0.4	1.57	±	0.05	1.506	±	0.004	7.14	±	0.05

The much higher Young’s modulus of thinner specimens was explained by heterogeneity in the tensile direction. It was concluded that the surface directly in contact to the mold was different from the inner part of the material. Reducing the mass charged in the mold was believed to produce specimens with the same skin and skin width but lower core width. Therefore, the variation of mass actually changed the skin/core ratio in the compressed sample. This skin-effect was mentioned previously on natural fibers moldings [6]. The skin of our specimens possessed a much higher Young’s modulus than the core, but did not bring additional strength, thus it was thought that the molecular organization may be different for the α-cellulose directly in contact to the mold, probably because the temperature was higher on the surface due to low thermal conductivity of cellulose.

### 2.3. Effect of Initial Moisture Content

It was commonly thought that the self-bonding mechanism of cellulose is based on the creation of inter-molecular hydrogen bonds. This statement was discussed recently and contribution of hydrogen bonding might after all have been overestimated [23]. Also, a mechanism known as Hornification, described by Fernandes Diniz *et al*., may lead to reduction of the bonding ability of cellulose, which happens when the fibers are dried over a certain point [24]. 10 grams of dried (0% moisture content), 6.1%, 8% and 10.8% moisture content α-cellulose (Table 2—Experimental section) were compressed using the control press cycle (Figure 13—Experimental section).

**Table 2:** Moisture content of α-cellulose samples obtained from equilibrium (2 weeks) at different relative humidity.

Temperature (°C)	Relative Humidity (%)	Measured Moisture Content (%)
103	0	0
25	45	6.1 ± 0.1
25	60	8.0 ± 0.0
25	75	10.8 ± 0.1

**Figure 3:**
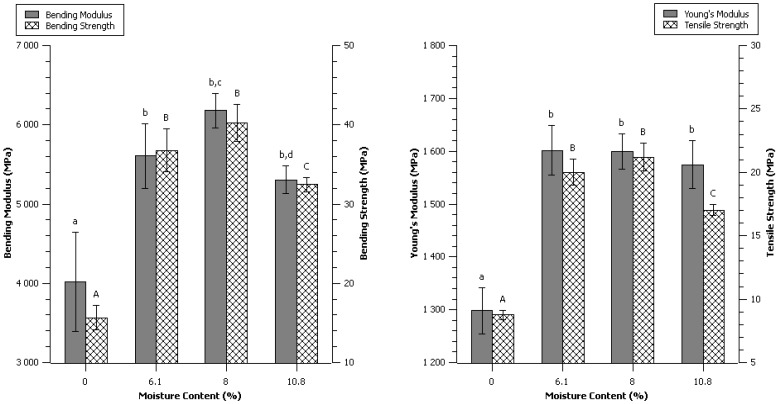
Effect of initial moisture content. Data are given as mean ± confident interval (95%). The letters on top of the column express significant difference of means according to Scheffé test with a 95% confident level. Small letters for moduli and capital letters for strengths.

The aspect of the dried compression molded α-cellulose was quite different from the others. The surface was rough, looking less glossy and the thickness of the specimen was much higher. The mechanical properties were also much lower on every parameter. Considering bending, a maximum was clearly observed around 8% of moisture content (Figure 3). Higher or lower moisture content led to a decrease of the mechanical properties. Similar observation was made for the tensile strength, but could not be observed on the modulus. Relatively high scattering could hide a more complex behavior but with these results no precise role of water content on the Young’s modulus could be affirmed, only the absence of water was concluded to be prejudicial.

### 2.4. Effect of Pressing Temperature

The pressing temperature ranging from 100 °C to 200 °C was investigated. 200 °C specimen exhibited delamination for about one third of the specimens. The other specimens did not show any defect or observable difference of visual aspect.

**Figure 4:**
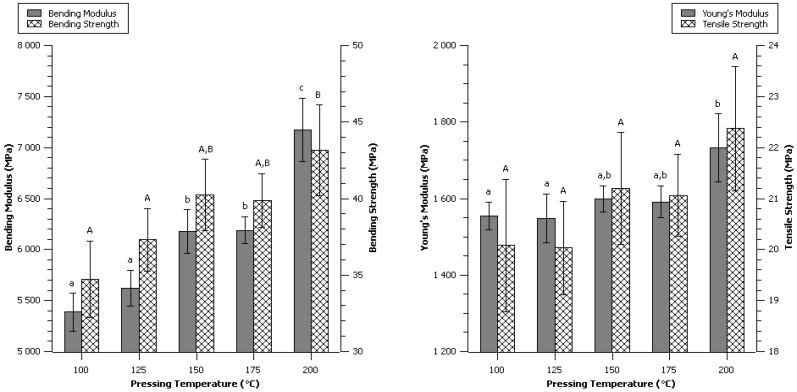
Effect of temperature during the compression process. Data are given as mean ± confident interval (95%). The letters on top of the column express significant difference of means according to Scheffé test with a 95% confident level. Small letters for moduli and capital letters for strengths.

No significant difference was observed on the tensile strength, and concerning the Young’s modulus, only 200 °C was significantly higher than 100 °C and 125 °C specimens (Figure 4). As far as bending tests are concerned, an obvious and highly significant effect of the temperature was observed to increase both bending strength and bending modulus.

Higher temperatures seem to alter more bending properties than tensile. The three-points bending properties are more representative from the top and bottom surface of a specimen; therefore, temperature was concluded to have a more drastic effect on the surfaces. High temperature of molding led to stronger, more brittle material. The effect of moisture content on the mechanical properties was previously seen to be important (Figure 3), it is highly probable that the interdependent relationship between temperature and water content might have major impact on the self-binding of α-cellulose.

The 200 °C condition produced the best specimens overall which exhibited a bending strength of 43.2 ± 3.0 MPa, a bending modulus of 7.17 ± 0.31 GPa, a tensile strength of 22.4 ± 1.2 MPa and a Young’s modulus of 1.73 ± 0.09 GPa (Table 1).

### 2.5. Effect of Pressing Pressure

The effect of twice lower pressure and combination of low pressure and low temperature of 100 °C was investigated with all other parameters unchanged.

**Figure 5:**
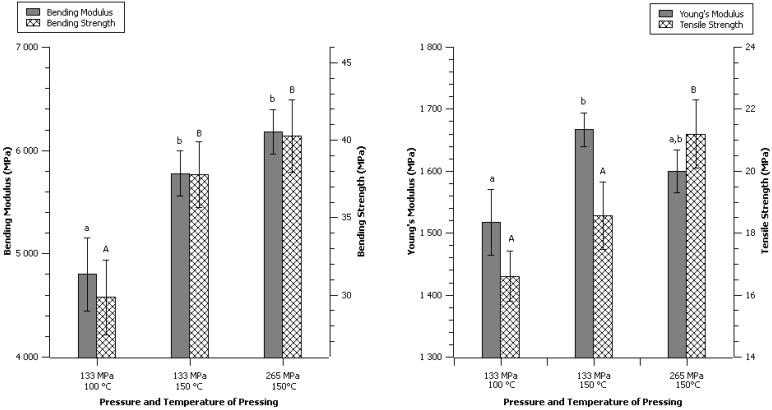
Effect of compression pressure and low temperature. Data are given as mean ± confident interval (95%). The letters on top of the column express significant difference of means according to Scheffé test with a 95% confident level. Small letters for moduli and capital letters for strengths.

The lower pressure showed significant difference from the control only for the tensile strength (Figure 5). The combination of low pressure and temperature did exhibit significant difference for all parameters except for the Young’s Modulus. The pressure, in this range from 133 to 265 MPa seemed to have a lower impact than the temperature.

### 2.6. Effect of Pressing Time

Some reports dealing with the production of binderless particle board had long time of compression-molding from 10 min (Okuda *et al*. [6] 2004) to 20 min (Shen [25] 1986, Hashim *et al*. [26] 2010). On the contrary, the process of pharmaceutical tablets production uses very short times of pressing, more like a single punch or impulsion [22]. This study on α-cellulose explored the effect of time at maximum pressure from 3 to 300 s, all other parameters remained unchanged from control condition.

**Figure 6:**
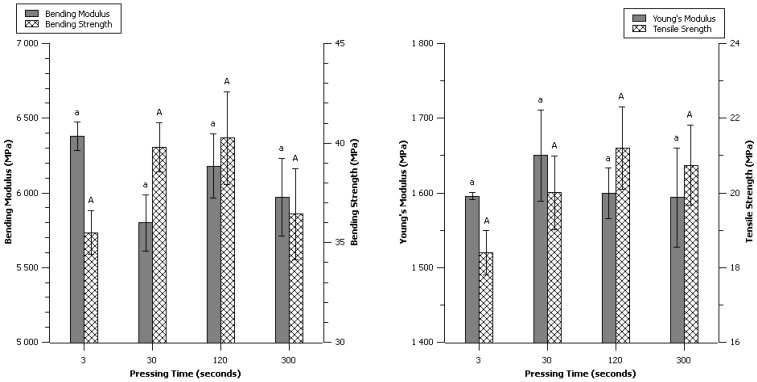
Effect of pressing time: Data are given as mean ± confident interval (95%). The letters on top of the column express significant difference of means according to Scheffé test with a 95% confident level. Small letters for moduli and capital letters for strengths.

No significant differences were observed from 3 to 300 s of pressing as seen on Figure 6. The possibility of compression molding cellulose or natural fibers in very short times was a very surprising and encouraging statement for potential industrialization adoption. Nevertheless, the bending and tensile strengths at 3 s and 300 s looked lower than the others, even if the scattering made this difference not significant according to statistical analysis. It is possible that a little dependence existed between time and mechanical properties, a certain time might be necessary to achieve highest mechanical properties, and thermal degradation may occur at longer times of pressing thus reducing the mechanical properties.

### 2.7. Effect of Pressure Establishment Rate

The pressure establishment rate is a very difficult parameter to investigate, because changing this rate also changes the total time of pressing or the pressure itself. It was chosen to maintain constant the integral “pressure time” thus adjusting the time of pressing while maintaining a constant final pressure (at its maximum) (Table 3).

**Table 3:** Time, Pressure and cycle of the Pressure rate increase series of experiments.

Prate	I pressure incrase phase (bar.s)	I plateau at max pressure (bar.s)	I (bar.s)	Time on pressure increase phase (s)	Time on plateau at max pressure (s)	Max hydraulic pressure reached (bar)	Max process pressure reached (Mpa)
10 bar/s	4500	36,000	40,500	30	120	300	265
50 bar/s	900	39,600		6	132	300	265
2 bar/s	22,500	18,000		150	60	300	265
1 bar/s	40,500	0		285	0	285	252

**Figure 7:**
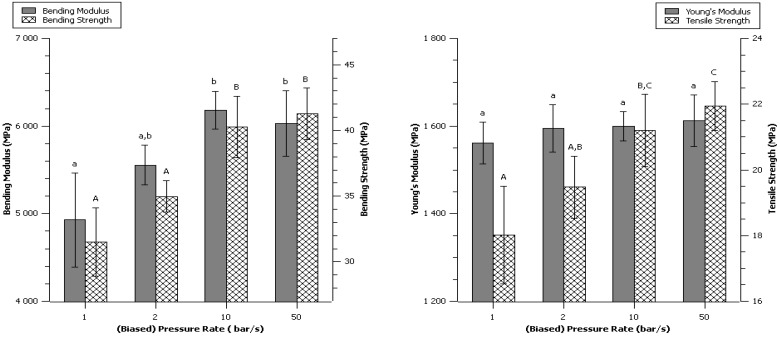
Effect of pressure establishment rate. Data are given as mean ± confident interval (95%). The letters on top of the column express significant difference of means according to Scheffé test with a 95% confident level. Small letters for moduli and capital letters for strengths.

Very good values were obtained with very high pressure establishment rate of 50 bar/s (maximum on the press), so a harsh pressure increase at the beginning of the cycle was not prejudicial for the mechanical properties (Figure 7). Akande *et al*. [22] studied the compression speed of 1:1 paracetamol : microcrystalline cellulose in the context of the pharmaceutical tablets production, it was reported that increasing the compression speed from 78 mm/s to 400 mm/s lowered the mechanical strength, but the conditions were so different that comparison was not thought to be relevant. Compression speed is however an important parameter to study and has great importance in the compression molding of pharmaceutical tablets.

The Young’s modulus was not impacted by the different conditions; these results are consistent with results obtained on pressure (Figure 5) and time (Figure 6) which had separately no impact on the Young’s modulus.

The other parameters were affected positively by the increase of the pressure rate. Time (Figure 6) had little impact on these parameters, and so did pressure from 133 to 265 MPa (Figure 5). Setting up a low pressure rate induced a lower pressure and a higher time, conditions that were separately leading to lower mechanical properties. Therefore, the weak properties produced by a low pressure rate could simply result in the combine effects of higher time and lower pressure. The slight improvement from control to 50 bar/s made think that pressure rate had a positive impact although the experimental design could not isolate this parameter. The suggested reason was that a fast application of the pressure did not let much time to water to move, therefore on the skin of the material, high pressure and high temperature were applied with very little time for the surface to dehydrate, which may improve the quality of the skin.

## 3. Discussion

A first statement of this study was the possibility of molding cellulose objects with interesting mechanical properties in a very short time. Yano *et al*. [15] used a days-long drying step and a further 30 min of pressing to obtain very high performance nanofiber sheets. Later, Iwamoto *et al*. [16] suppressed the pressing step but needed 48 h at 55 °C and final drying at 105 °C to obtain the material. Nogi *et al*. [17] confirmed this process when they first introduced their optically transparent nanofibers paper which was obtained by a 72 h drying step. Very long processing times are also observed in the case of solubilization of cellulose for obtaining “all cellulose composites” [13,14].

In the case of binderless particleboards, long processing times are expected when directly pressing raw material usually 10 [6] or 20 min [26]. When the starting material is pre-treated using steam explosion, molding times can be reduced, e.g., 80 s [3].

In this study, three seconds of compression molding without using any pre-treatment to fibrillate the cellulose gave specimens with decent mechanical properties: 35.5 ± 1.1 MPa of bending strength, 6.38 ± 0.10 GPa of bending modulus, 18.4 ± 0.6 MPa of tensile strength and 1.60 ± 0.00 GPa of Young’s modulus (Table 1). These mechanical properties were not able to compete with the highly refined nanofiber cellulose papers: our specimens had about five times lower properties in bending [15], and even 20 times in tensile considering the composites obtained by cellulose dissolution [13] but will be sufficient in most of common applications. The absence of pre-treatment for defibrillation is therefore thought to be the most probable explanation for the much lower performances observed in this study compared to the others. Anyway, decent mechanical properties obtained in a very short processing time make it possible to consider an industrialization of this process.

It is commonly thought that density on natural fiber binderless boards brings higher mechanical properties as demonstrated by Okuda *et al*. [6,27] and Widyorini *et al*. [8], for examples. The higher density of matter means that particles are being forced to be very close therefore increasing surface contact and the possibilities of producing hydrogen bonds and increasing van der Waals forces. Even Yano *et al*. [15] in the nanofiber paper field compared the density of the material obtained from original Kraft pulp (1.25 g/cm^3^) and highly refined fibrillated nanofibers (1.48 g/cm^3^), and it was reported a five times increase of the MOE and MOR with increasing the density.

**Figure 8:**
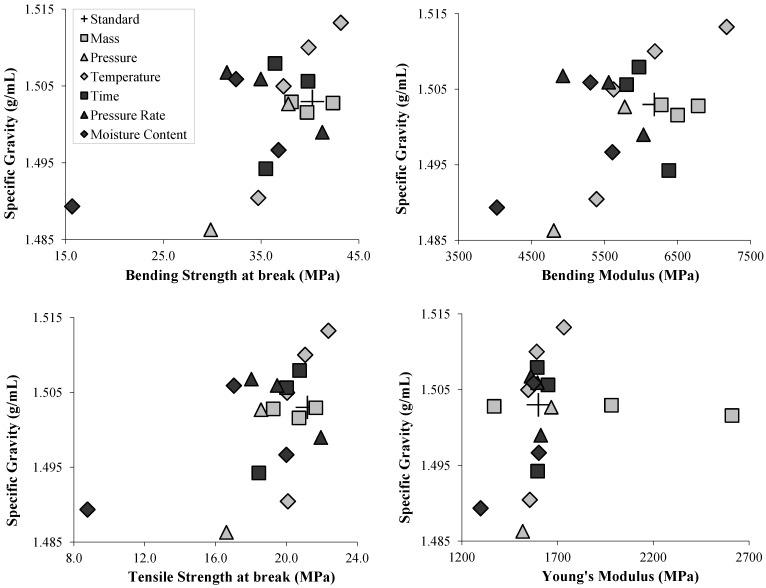
Specific gravity expressed against Tensile and Bending Strength at break, Bending Modulus and Young’s Modulus.

Generally in this study, a positive correlation was observed (Figure 8) between specific gravity and the mechanical properties but some points were out of the trend. Particularly, the parameter "Mass" showed no correlation, especially for the Young’s modulus, which drastically increased when reducing the mass, although specific gravity remained the same.

Because of the use of high pressure, every specimen showed a higher density ranging from 1.486 ± 0.007 to 1.513 ± 0.008 g/cm^3^. Nilsson *et al*. [19] obtained densities from 1.25 to 1.35 g/cm^3^ and Zhang *et al*. [21] reported density going from 1.42 to 1.52 on compression-molded cellulose which was close to the density of 1.599 for the cellulose crystal stated by Sugiyama *et al*. [28]. Comparing with the nanofiber paper field, densities are reported from 1.30 g/cm^3^ [16] to 1.48 g/cm^3^ [15]. Our measurements showed quite high densities for cellulosic materials, in the range of what was reported with compression molding or other techniques of casting and drying. With the much lower properties obtained with our specimens, it can be confirmed that the characteristics of the starting material and precisely the refining are playing a predominant role [15].

**Figure 9:**
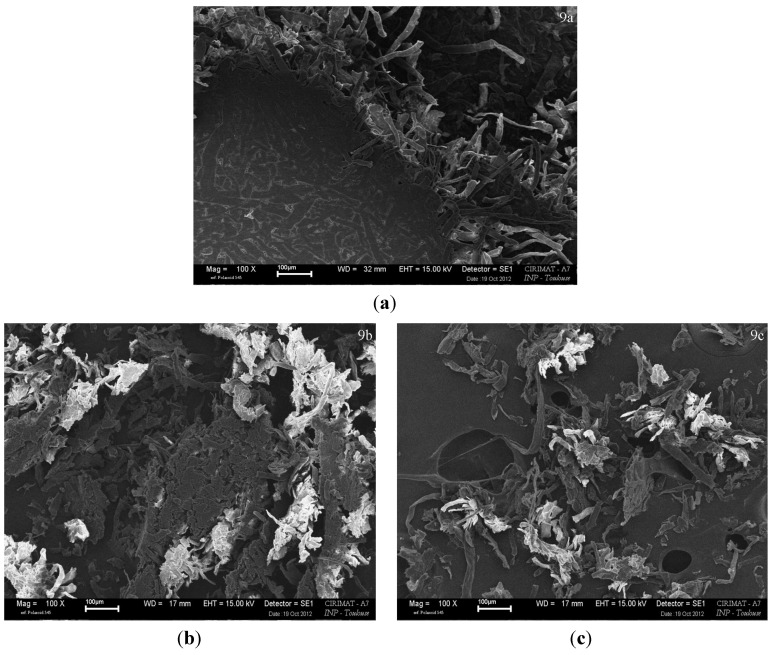
(**a**) Surface fracture after tensile break of a specimen (standard) In the lower left corner is the intact surface, and on the opposite appears the inner structure of the material; (**b**) 2 μm powdered surface of a specimen produced under standard conditions; (**c**) 2 μm powdered core of a specimen produced under standard conditions.

SEM observations were performed on the material itself, and a very compact network of fibers was observed on the surface of the material (Figure 9a). On some points, the limit between two or more fibers faded, and it was not possible to tell if it was one single ramified fiber or different fibers with their contacts zone “melted” one in the others. Nilsson *et al*. [19] used CP/MAS ^13^C NMR to get an indication on the size of the fibril aggregates in their compressed cellulosic material, they reported that the size of the aggregates increased with temperature, showing that fibrils could aggregate one to each other. This phenomenon of fibrils aggregation could be linked to what happens during the hornification of cellulose fibers, which possible explanation was reported by Newman [29] to be co-crystallization.

On the fracture (Figure 9a) the inner part exhibited a less compact structure, with almost “free” fibers. In the inner part, the compact structure observed on the surface either did not exist or was destroyed during the breaking. The surface (Figure 9b) and inner part (Figure 9c) of one control specimen were powdered using a microtome. Blocks of packed fibers were cut out of the surface by the microtome, although in the inner part, much more free fibers were cut out by the microtome. The packed fibers cut out from the surface were considered as an indication for a higher degree of organization of this surface, compared to the core from which fibers were separated much more easily.

This organization observed on the surface (Figure 9a,b) might be due to the higher temperature on the surface of the material, which was in direct contact with the mold. Also it has to be considered that higher temperature of molding increased (Figure 4) the mechanical properties and that surface was confirmed to possess a higher Young’s modulus (Figure 2). The specific gravity and moisture of this skin seem not to be different from the inner part of the material because specific gravity of specimen produced with different initial masses (different ratios surface/core) had similar specific gravities (Table 1).

Equilibrium moisture content at 60% relative humidity and 25 °C was measured as a measure of the overall hygroscopy of the specimens, indicating the amount of accessible hydroxyl groups.

**Figure 10:**
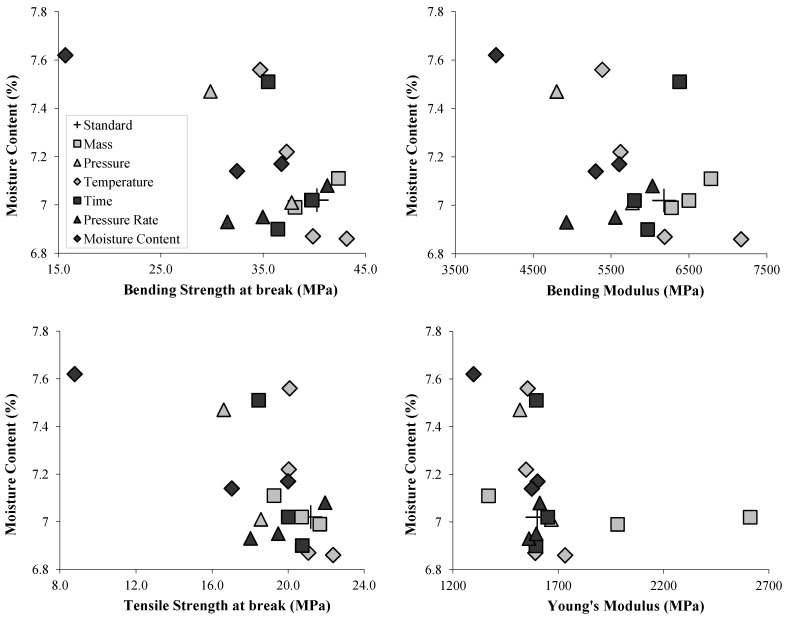
Moisture content of compression-molded specimens at 60% RH expressed against Tensile and Bending Strength at break, Bending Modulus and Young’s Modulus.

Nilsson *et al*. [19] reported measuring less accessible cellulose fibrils when increasing the temperature of molding. Our measurements are in accordance to their observations as the moisture content of higher temperature molded specimens is decreasing with temperature (Table 1), showing that the fibrils are less accessible to atmospheric water vapor. For the other parameters, the correlation between moisture content and the mechanical properties was not obvious: specimens with different masses introduced in the mold had very close moisture contents (Figure 10). In addition, the lowest time of molding produced weaker specimens with higher moisture content, which followed the trend, but highest time produced drier specimens with slightly weaker mechanical properties than the control. Thermal degradation and the shortening of the cellulose chains [30] could be an explanation for this, although not entirely satisfactory because of the good mechanical properties obtained at 200 °C and the comparison with other molding times (Yano *et al*. [15] 30 min at 150 °C, Nilsson *et al*. [19] 20 m between 120 °C and 180 °C, Rampinelli *et al*. [20] 6 min at 160 °C) which were much longer than ours however with better mechanical properties.

**Figure 11:**
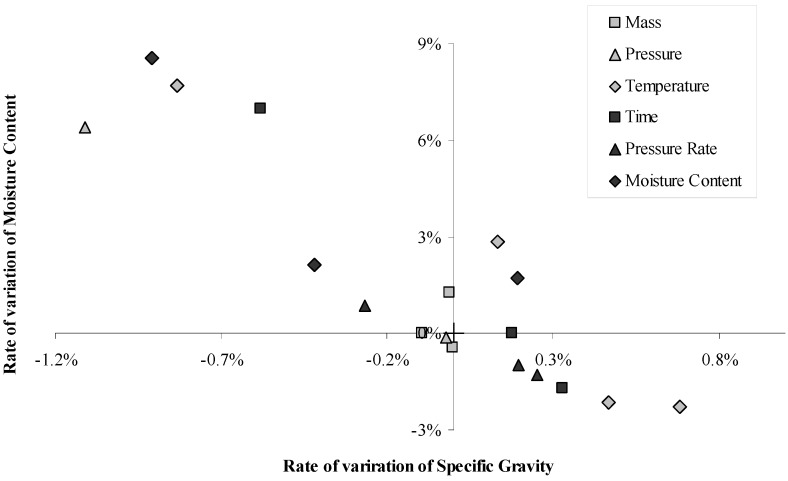
Rate of variation of moisture content *versus* rate of variation of specific gravity. Rates of variation calculated comparing each modality to the control conditions.

Figure 11 shows that specific gravity and moisture content were satisfactorily correlated, although the mechanical properties, representative of the bonding, could not be satisfactorily correlated to either specific gravity or moisture content, the mechanisms of bonding must therefore be complicated and imply several phenomena.

Molding dried alpha cellulose produced specimens with much lower mechanical properties, but specific gravity (1.489 ± 0.004 g/cm^3^) and moisture content (7.62% ± 0.02%) were comparable to other conditions e.g., 3 s of pressing time (1.494 ± 0.005 g/cm^3^ and 7.51% ± 0.04%), low temperature (1.486 ± 0.007 g/cm^3^ and 7.56% ± 0.06%). Hornification [24,29] may have occur when drying the starting material, with irreversible changes preventing it from producing decent performances specimens when being processed.

The moisture content of the resulting specimen was however lower (7.62% ± 0.02%) than starting material α-cellulose (8.0%) showing that the fibers were less accessible to atmospheric water vapor on a macroscopic point of view. The evaluation of what was really happening to the fibers during the molding would have required the surface and porosity analysis, which were not performed here. Hydrogen bonding and other types of interactions which were discussed recently could occur between cellulose chains as well as co-crystallization [29].

## 4. Experimental Section

### 4.1. α-Cellulose Compression-Molded Specimen Production

α-cellulose (96% purity according to the provider, with 4% insoluble hemicelluloses [31]) was purchased from Sigma-Aldrich (St Louis, MO, USA) under the reference “bulk cellulose”, product was extracted from aspen trees. The crystallinity index has been measured at 62%. The crystallinity index of starting material was measured using X-ray diffraction. It was performed on a quartz-lead sampler loaded with the α-cellulose powder using a MiniFlex II Desktop X-ray Diffractometer with Cu Kα radiation (Rigaku ,Tokyo, Japan). Scans were obtained from 5 to 50 degrees 2θ in 0.05 degree steps for 15 s per step. The crystallinity index was calculated by a method developed by Segal *et al.* [32] and widely re-used since then.

Samples with different moisture contents were generated by letting α-cellulose at least two weeks to equilibrate with different controlled atmospheres at different relative humidities using a Climacell climatic chamber (Fisher Scientific, Bioblock Scientific, Illkirch, France).

Moisture content of each sample was measured in triplicate by comparing a sample's mass to its dry basis which was obtained in 48 h at 103 °C in an oven (Memmert Gmbh, Schwabach, Germany, model 600). Values are presented in table 1.

**Figure 12:**
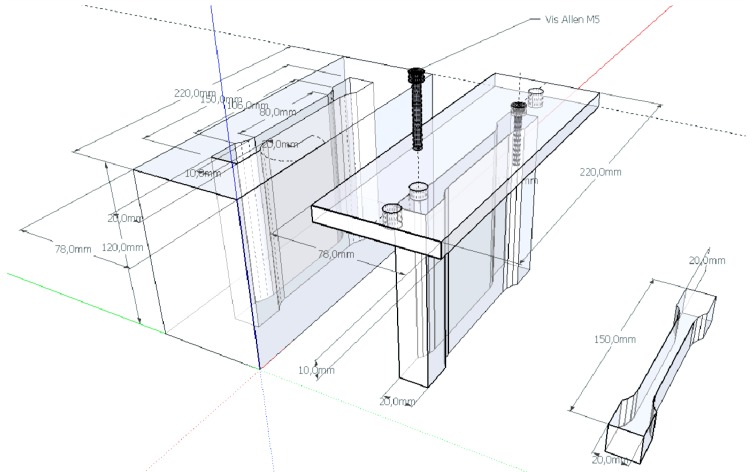
NF EN ISO 3167 1A specimen compression mold: schematics.

A mold was custom-machined (Cristin Electro Erosion, Grisolles, France) in order to produce 1A dog-bone tensile specimens according to ISO 3167 standard [33], it consists in a die, a stopper at the bottom, and a punch (Figure 12). The thickness of the samples is conditioned by the mass of matter introduced in the mold and apparent density of the final product, the average thickness obtained was 3.66 mm when 10 g of matter were charged in the mold which was little bellow the 4 mm required by the standard.

The mold was placed in a computer-controlled laboratory-scale hydraulic press (Pinette Emideceau, Chalon sur Saône, France), capable of generating a maximum of 530 kN of effort (about 50 tons). The metal plates were thermo regulated, and used to warm up the mold at least 30 m before each molding attempt. The temperature of the mold was verified to be the same as the plates with an infrared temperature sensor.

The α-cellulose powder was pre-weighted and taken out of the climatic chamber in a closed container to maintain moisture content as much as possible. It was charged in the mold manually, as quickly as possible to prevent change of the moisture content, and great attention was paid at trying to get a homogeneous repartition.

A control press cycle and conditions were determined, based on preliminary results. It was found that 10 g of matter, equilibrated at 60% relative humidity, compression-molded at 150 °C for 2 min was producing satisfactory pieces in terms of finish, touch, mechanical resistance, and they were intact although some other conditions made them explode. These were defined as the control conditions. Different series of experiment with various parameters were performed, in order to investigate the effect of Mass, Temperature, Pressure, Moisture Content, Time and Pressure establishment Rate on the mechanical properties of the resulting specimens (Table 1). The process pressure was calculated with the force measured with a dedicated sensor. 265 MPa corresponded to the maximum load (50 tons) of the press.

**Figure 13:**
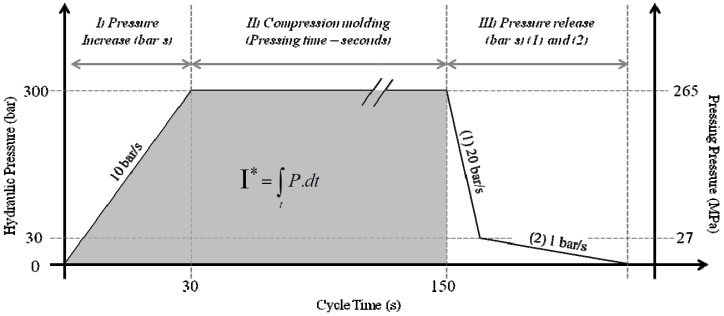
Typical press cycle for control conditions. The investigation of Time used different time of phase II. The investigation of Pressure Rate Increase impacted phase I. The integral I was taken constant for the Pressure Rate investigation series.

For the investigation of the pressure rate increase influence in the process, the integral I (Figure 13) was calculated on the control. Pressure rate was set up at 1 bar/s, 2 bar/s, 50 bar/s (hydraulic pressure) while adjusting Time to maintain equal I. Therefore, the time was different for each experiment, but also the maximum pressure of 265 MPa was not even obtained for the 1 bar/s experiments (Table 3).

### 4.2. Tensile and 3 Points Bending Tests

Tensile and 3 point bending tests were conducted according to ISO 527-2 [34] and ISO 178 [35] using a H5KT Universal testing machine (Tinius Olsen, Horsham, PA, USA) at 1mm/min speed. These standards are related to the evaluation of mechanical properties of plastics.

From these tests, four variables were collected: Young’s Modulus, Tensile Strength at break, Bending Modulus and Bending Strength at break. Significance of difference between means of the 4 variables was analyzed with ANOVA and Scheffé’s contrasts test (multiple comparison of means) using Matlab (Mathworks, Natick, MA, USA). Every statistical analysis was performed with 95% confidence. The tests were performed within each series of experiment, and not on the whole set of experimental results.

Some specimens were presenting defects like cracks when compression molded at 200 °C, or sometimes heterogeneity marked by lighter spots where density seemed lower. These “low density” spots were probably generated when charging the material in the mold inhomogeneously. These specimens were marked and their results were not taken into consideration. Seven to 15 repetitions were done on each condition, the obtained specimens were let to equilibrate for two weeks at 25 °C and 60% of relative humidity before mechanical tests.

The confident interval (1) of the mean was calculated from the standard deviation:

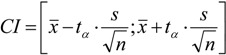
(1)
where *x* bar is the estimated mean; *t_α_* a coefficient resulting from the Student law (1.96 for a 95% confidence); *s* the standard deviation of the series, and ***n*** the number of specimen within the series.

### 4.3. SEM Observations

Control sample material was observed by scanning electron microscopy: LEO 435 VP microscope, Leo Electron Microscopy Ltd (Cambridge, UK) sample silver plating before observation. One control specimen was observed on the fracture after a tensile test. The surface and the core of another standard specimen were separated and very thin shavings of 2 μm were cut out of surface and core using a RM2125RT microtome (Leica Microsystems, Wetzlar, Germany) equipped with a tungsten blade. The two powders (surface and core) were observed.

### 4.4. Specific Gravity and Moisture Content of the Specimen

Six 2 cm long pieces were cut out of six different dog-bone specimens (from the mechanical tests) to measure specific gravity and moisture content of the materials. These six small pieces were first let to equilibrate in a 60% relative humidity 25 °C climatic device, room and moisture content was measured on 103 °C oven-dry basis.

The dried 6 pieces were put back in the oven and then used for evaluation of specific gravity using a YDK01 Density determination kit on a Sartorius MC210P precision scale (Sartorius AG, Goettingen, Germany). The specimen were weighted in the air, and then dipped into cyclohexane (Sigma Aldrich, St Louis, MO, USA, Chromsolv for HPLC purity > 99.7%) and the resulting Buoyancy (W_air_ − W_fluid_) was measured. Specific gravity was measured using (2):

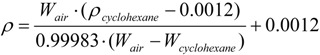
(2)
where ***ρ*** is the specific gravity of the materials tested; *W_air_* and *W_cyclohexane_* the weight of the tested specimen in the air and in the cyclohexane respectively; *ρ_cyclohexane_* the specific gravity of cyclohexane at the measured temperature during the experiment; 0.0012 (g/cm^3^) the specific gravity of air at 20 °C and 101.325 kPa; and 0.99983 a manufacturer’s correction factor related to the increase of the fluid level and immersion of the wires in the fluid when the specimen is dipped in the fluid.

Cyclohexane was chosen arbitrary, air bubbles were chased out of the material when immersing it in the solvent, but some of the porosity of the material might remain inaccessible to the solvent, so the specific gravity measured here may differ from real absolute value of density. However, it is thought to be close to the real density and significantly higher than the apparent density.

Measuring dried specimen was thought to prevent misevaluation of specific gravity due to difference of water content between the specimens. On the control specimens, the measured specific gravity of the material equilibrated at 60% relative humidity and 25 °C was 1.485 ± 0.003 g/cm^3^. On the other hand, the measured specific gravity of the dried material was 1.503 ± 0.002 so the measurements on the dry matter over-estimated.

## 5. Conclusions

Compression molding in a hot press was successfully performed with pure α-cellulose. The specimens exhibited a smooth, plastic-like surface. The best conditions (200 °C, 265 MPa, 120 s, 8% moisture content) produced specimens with a bending strength of 43.2 ± 3 MPa, a bending modulus of 7.17 ± 0.31 GPa, a tensile strength of 22.4 ± 1.2 MPa and a Young’s modulus of 1.73 ± 0.09 GPa. Under these conditions, delamination could occur showing how precise the adjustments of the parameters must be. It appeared that these extreme conditions where specimens can explode due to steam accumulation are the ones that produced the best specimens. These properties are comparable or higher than conventional petroleum-based glassy plastics.

The effects of several parameters were investigated to study the effect on the mechanical properties of pure α-cellulose compression molded specimens. It was observed that moisture content and temperature were the most important parameters. The effects of time and pressure were not significant in the considered ranges. The moisture content and specific gravity of the resulting specimens decreased and increased respectively with the mechanical properties for the Temperature and Moisture content. A correlation was observable only for those two parameters but not on the others. The presence of water in the starting material was demonstrated to be crucial in order to elevate the mechanical properties.

The mass introduced in the mold was a significant parameter that revealed a skin-effect. The skin of the material showed a much higher Young’s modulus than the core of the material, but other parameters were similar in the skin and the core. This statement is calling for more investigations.

Very short times of three seconds of molding were observed to produce correct specimens with slightly lower strengths. The very short time of processing was an encouraging statement to consider a possible future industrialization of the process.
